# ^82^Rb PET imaging of myocardial blood flow—have we achieved the 4 “R”s to support routine use?

**DOI:** 10.1186/s13550-016-0225-4

**Published:** 2016-09-20

**Authors:** Robert A. deKemp, Ran Klein, Rob S. B. Beanlands

**Affiliations:** 1 Division of Cardiology, National Cardiac PET Centre, University of Ottawa Heart Institute, Ottawa, Canada; 2 Division of Nuclear Medicine, The Ottawa Hospital, Ottawa, Canada

**Keywords:** Rubidium, Rigorous validation, Repeatability, Reproducibility, Reliability, Robustness, Revascularization therapy, Resource efficiency

Routine quantification of myocardial blood flow (MBF) in absolute units of mL/min/g has long been one of the technical milestones expected to enable the widespread clinical application of cardiac positron emission tomography (PET). The recent study by Germino et al. [1] contributes significantly to the increasing body of literature supporting the potential for rubidium-82 (^82^Rb) PET to become the de facto clinical standard method for non-invasive quantification of MBF and myocardial flow reserve (MFR) in the routine diagnosis and management of patients with ischemic heart disease. The authors are commended for completing a very technically challenging study, including three-dimensional PET parametric imaging and arterial blood sampling validation with the ultra-short-lived tracers ^82^Rb and ^15^O-water.

^82^Rb is currently the most widely used tracer for PET myocardial perfusion imaging (MPI) in North America, and its use is increasing in Europe and in other parts of the world. While the diagnostic accuracy of ^82^Rb PET for routine MPI is accepted to be slightly higher than ^201^Tl or ^99m^Tc-based SPECT methods [2] and is recommended over SPECT in certain patient sub-groups [3], the full added value of PET for MBF quantification has yet to be realized in improving patient management to reduce adverse cardiac outcomes such as myocardial infarction and death. In the present issue, Germino and co-authors have solidified the foundation upon which future clinical trials can be performed to help achieve this long-term goal. To have full confidence in MBF PET imaging results sufficient to direct optimal therapies in the clinical routine, the following criteria should be demonstrated.

## Rigorous validation (accuracy)

The tracer kinetics of ^82^Rb were first shown in rabbits to follow a two-compartment model [4], but a simpler one-tissue-compartment model (1TCM) has been shown to be adequate to describe the typical kinetic profiles in human and animal model studies [5]. This 1TCM has only three parameters, which Germino et al. used successfully for the estimation of parametric images showing the influx-rate (K1 uptake), efflux-rate (k2 washout), and fractional blood volume (V_A_ spillover). MBF values are derived from the uptake-rate K1, using a tracer extraction function E(MBF) calibrated to an accepted gold-standard. In experimental animal studies, ex vivo quantification of radioisotope- or fluorescent-labeled microspheres has been used to validate dynamic PET MBF measurements using ^15^O-water and ^13^N-ammonia, both of which are (nearly) freely diffusible across capillary and cell membranes [6, 7]. Because the microsphere technique cannot be used in humans, the 1TCM method for ^82^Rb MBF was validated originally against ^13^N-ammonia in healthy normal subjects and heart disease patients using 3D dynamic PET and filtered-back-projection analytic reconstruction [8]. The estimated extraction function was then confirmed with ^15^O-water measurements using 2D dynamic PET and iterative reconstruction [9]. These findings are now re-confirmed by Germino et al. using state-of-the-art 3D PET-CT parametric imaging with iterative time-of-flight reconstruction and detector response modeling. There are several important findings in their study that highlight the need for attention to the methodological details, in comparing or standardizing ^82^Rb MBF measurements between different imaging laboratories.

As in a previous study by Weinberg [10] ^82^Rb arterial blood samples were compared to the PET image-derived blood input functions (IDIF). In the present study, Germino et al. found a small under-estimation (−3 and −8 %) in the ^15^O-water and ^82^Rb IDIF values, that was not time-dependent, and could therefore be corrected by simple scaling. The authors propose that the source of this small bias may be related to technical factors, e.g., positron range or other resolution effects, but may also be attributed to physiological factors, e.g., red blood cell uptake which has been demonstrated with other potassium analogs such as ^201^Tl [11]. Similar to ^15^O-water, ^82^Rb does not bind to plasma proteins nor does it have any radioactive metabolites in arterial blood or myocardial tissue, which can complicate the tracer kinetic analysis of other MBF tracers such as ^13^N-ammonia [12].

As the source of the reported IDIF bias was not confirmed, the authors explain that the derived scale-factor may be “scan dependent, conditional on variations in VOI size and placement, heart size, breathing pattern, and subject motion. Further investigation is required to assess generalizability to other scanners and reconstruction algorithms.” However, it is important to note that regardless of the cause of the apparent bias, when the measured ^82^Rb IDIF values were used without the scaling correction, the corresponding extraction fraction parameters were very similar to those reported by Lortie [8, 9].

**Fig. 1:**
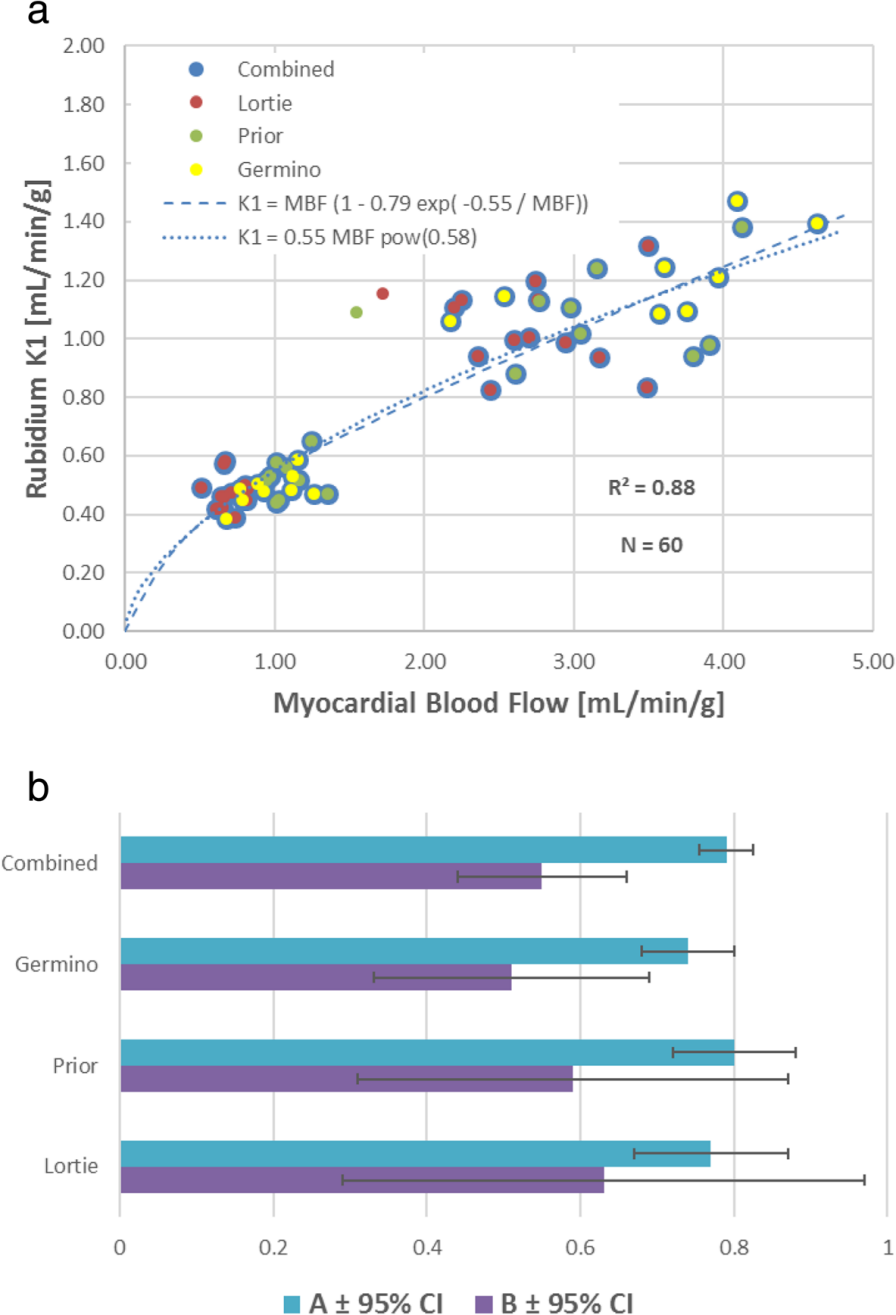
**a** Combined estimation (*N* = 60) of ^82^Rb extraction function parameters from three validation studies performed in humans using the one-tissue-compartment model, without correction (spillover or scaling) of the arterial blood input function. **b** Combined estimates of the ^82^Rb extraction parameters: *A* (0.79 ± 0.035) and *B* (0.55 ± 0.11) demonstrate improved precision vs. individual studies (Germino, Prior, Lortie). Confidence interval (CI) values derived from Table 3 in Germino et al. [1]

By combining the new data from Germino et al. together with these two previous studies, which all used comparable implementations of the 1TCM (without any blood input function correction), we can improve the estimation of the ^82^Rb extraction function parameters (Fig. 1a), which in turn should improve the precision of the derived MBF estimates [13, 14]. Contrary to previous reports [15], these data clearly demonstrate that there is no “roll-off” of tracer uptake-rate using the 1TCM, as the K1 values continue to increase with MBF over the range of physiological values measured in these human studies. Interestingly, an empirical power function appears to describe the rubidium K1 vs. MBF relationship as well as the more physiological Renkin-Crone tracer extraction formulation. Recognizing that the fitted functions shown in Fig. 1a are intended primarily to “calibrate” the ^82^Rb uptake rates (K1) for accurate estimation of MBF, a strict physiological interpretation is not required. Therefore, it would seem appropriate to use a function similar to one that fits the “combined” data of these three validation studies. It is encouraging that these combined results seem to be relatively independent of the particular scanning hardware, acquisition protocol, image reconstruction, and software analysis methods used in the respective studies individually.

## Repeatability (test-retest and operator variability)

Several studies have demonstrated very good repeatability of measured MBF values using ^82^Rb dynamic PET, on the order of ±5 % limits-of-agreement when compared within or between operators Klein et al. [16] and ±20 % between sequential imaging sessions under both rest and hyperemic stress conditions [17–19]. Test-retest repeatability of ^82^Rb MBF was substantially higher (±35–40 %) when measured on separate days, and analyzed using a tracer retention model [20], which is a further simplification of the 1TCM that measures the net effects of tracer uptake and washout. MBF test-retest repeatability can be improved to ±10–15 % by using a standardized “square-wave” tracer infusion profile, available with the latest ^82^Rb generator system [21].

## Reproducibility (multi-center standardization)

The commonly applied 1TCM for ^82^Rb [8] does not require any a priori assumed methodological constants (e.g., tissue distribution volume, blood integration interval, myocardial wall-thickness, or partial-volume recovery coefficient) or physiological constants (except the ubiquitous extraction function), making the model widely applicable and reproducible despite local variations in PET scanner technology (e.g., 2D vs. 3D), dynamic sampling (2 to 10 min), image reconstruction (analytic vs. iterative ± time-of-flight or detector response modeling), and computer software implementations [22–25].

The three human ^82^Rb validation studies using the same 1TCM have produced consistent estimates of the extraction function parameters (Fig. 1b) with overlapping 95 % confidence intervals (CI). Murthy et al. [26] have also demonstrated similar prognostic value between 1TCM variant methods when stress/rest myocardial flow reserve (MFR) was used to predict patient adverse outcomes. Despite these significant advances, some challenges remain in terms of weight-based dosing to maintain MBF accuracy with early-generation 3D PET technologies [27, 28] and standardization of hyperemic stress response when using different pharmacologic stressors such as dobutamine, adenosine, adenosine triphosphate, dipyridamole, or regadenoson [29].

## Revascularization vs. medical therapy decisions (clinical and cost effectiveness)

As the availability of ^82^Rb continues to increase and standardization of PET MBF methods improves between imaging laboratories, the ability to conduct multi-center trials demonstrating the clinical value to direct effective therapies will improve accordingly. The cardiac PET community could strive to produce non-invasive imaging evidence similar to the pivotal FAME trial [30] which used invasive measurements of fractional flow reserve (FFR) to identify flow-limiting stenoses associated with myocardial ischemia, direct effective therapy, improve patient outcomes, and reduce treatment costs, leading to revised European Society of Cardiology guidelines for myocardial revascularization in patients with ischemic heart disease [31].

Johnson et al. [32] have proposed a stress PET MBF threshold ~0.9 mL/min/g to identify definite myocardial ischemia; however, the relative value of stress MBF vs. stress/rest MFR remains to be elucidated for the optimal detection of disease and management of therapy decisions [33]. In this regard, it is critical to understand the differences between the measurement of epicardial FFR vs. epicardial + microvascular MFR, as elegantly described by Johnson and Gould [34, 35]. The addition of CT coronary angiography or microvascular-specific tests of endothelial function may have a role to play in the differential diagnosis of macro- (CTA) vs. micro-vascular (PET) disease, which should be amenable to revascularization vs. medical therapies respectively [36]. Prospective studies are needed to evaluate the role of PET MBF and MFR to direct therapies such as revascularization. In the meantime current clinical trials such as the DEFINE-FLOW study which is using invasive measures of fractional and coronary flow reserve will help to define the potential clinical role of non-invasive PET MFR to complement invasive FFR measurements of the physiologic consequences of coronary atherosclerosis [37]. Limited US data available supports the cost effectiveness of ^82^Rb PET MPI when used consistently in patients with intermediate pre-test likelihood of disease [38]; comparable studies are still needed in the European setting, to establish the financial value or impact of ^82^Rb for conventional MPI and the potential added value of routine quantification of MBF.
